# Game-Theoretical Design of an Adaptive Distributed Dissemination Protocol for VANETs

**DOI:** 10.3390/s18010294

**Published:** 2018-01-19

**Authors:** Cristhian Iza-Paredes, Ahmad Mohamad Mezher, Mónica Aguilar Igartua, Jordi Forné

**Affiliations:** Department of Network Engineering, Universitat Politècnica de Catalunya (UPC), C. Jordi Girona 1-3, 08034 Barcelona, Spain; ahmad.mohamad@upc.edu (A.M.M.); monica.aguilar@entel.upc.edu (M.A.I.); jordi.forne@entel.upc.edu (J.F.)

**Keywords:** game-theory, vehicular Ad hoc networks, safety messages, video dissemination

## Abstract

Road safety applications envisaged for Vehicular Ad Hoc Networks (VANETs) depend largely on the dissemination of warning messages to deliver information to concerned vehicles. The intended applications, as well as some inherent VANET characteristics, make data dissemination an essential service and a challenging task in this kind of networks. This work lays out a decentralized stochastic solution for the data dissemination problem through two game-theoretical mechanisms. Given the non-stationarity induced by a highly dynamic topology, diverse network densities, and intermittent connectivity, a solution for the formulated game requires an adaptive procedure able to exploit the environment changes. Extensive simulations reveal that our proposal excels in terms of number of transmissions, lower end-to-end delay and reduced overhead while maintaining high delivery ratio, compared to other proposals.

## 1. Introduction

Driving a vehicle is one of the most hazardous human activities. More than 1.25 million people die each year in traffic accidents worldwide, according to the Global Road Safety Report 2015 [1] released by the World Health Organization (WHO). The report also criticizes the fact that only 40 countries in the world sell vehicles that meet their safety requirements. Intelligent Transport Systems (ITS) have been proposed with the goal of using advanced technologies to improve safety and efficiency of transport systems. In this line, Vehicular Ad hoc Network (VANET) is shown as a key component of the future ITS to support safety, traffic management, and user infotainment applications [2]. VANETs are a type of mobile ad-hoc networks (MANETs) in which nodes are vehicles forming self-organized networks without the requirement of permanent infrastructure. The VANET topology changes dynamically due to the high mobility of nodes. Thus, link breakages are frequent which makes VANET communications be challenging.

VANETs can support a number of applications, namely infotaintment, safety, etc. Currently, there is a need to support vehicular communication for applications such as safety messaging, traffic and congestion monitoring and Internet access. One of the most promising application of VANETs are safety applications. Approaching emergency-vehicle warning, post-crash warning, accident reporting, blind merge warning, and pre-crash sensing, among others, are effective applications for improving road safety. Safety applications usually rely on broadcast-based protocols. These protocols have the task of disseminating emergence messages quickly and efficiently through the network. Hence, a key research problem here is how to design a scalable information dissemination method that can efficiently work with high reliability and short delay under different network conditions.

Most of the literature in this area aim to solve the broadcast storm and intermittently connected network problems for vehicular ad hoc networks where the high mobility of vehicles, unstable link connectivity, fading of signal, and obstacle-constrained environments are the major concerns on the data delivery performance. Beaconing or one-hop broadcast is one of the most common techniques for data dissemination in vehicular networks. Exchanging beacon messages is important for road safety applications. However, channel load may increase too much in scenarios with high vehicle density when the beacon rate is fixed. Within this mindset and assuming decentralized knowledge of the topology, efficient broadcasting could be formulated as a classical constrained optimization problem with the objective of minimizing the number of re-transmissions while at the same time guaranteeing high delivery ratio.

In this work, we propose an Adaptive Distributed Dissemination (ADD) protocol to perform data dissemination in VANETs. ADD is designed to operate without any roadside infrastructure in urban scenarios under diverse road traffic conditions. To achieve this objective, ADD employs a decentralized stochastic solution for the broadcast data dissemination problem through two game-theoretical mechanisms. Game theory can be used to design a mechanism to predict behavior in situations where a state is the result of a series of interactions between different nodes (refered as *players* in the game), who act according to their preferences regarding future performance and existing incentives. First, the Asymmetric Volunteer’s Dilemma Game modeled by Diekmann [3] is evaluated as a mechanism to quench the broadcast storm problem. The probability that a node forwards a broadcast message is calculated using the number of candidate vehicles to forward the message, i.e., the number of vehicles that are listening to the transmission. The cost/benefit relation to forward the message by the vehicle is obtained from metrics such as distance and link quality. Next, the Forwarding Game modeled by Naserian [4] is evaluated as another mechanism to mitigate the broadcast storm problem. In this case, the strategy of the players is to select a forwarding probability that maximizes their pay off using a utility function. The utility function is designed as a function of the player’s availability and the forwarding probability of other players. Availability of a player is a normalized factor based on metrics such as distance from the source of the flooding packet (e.g., an accidented vehicle) and estimated bandwidth of the link formed between the node currently holding the packet and each candidate node within its transmission range. Because exchanging vehicle information via beacon messages is important for active safety applications, our proposal includes the adaptive traffic beacon (ATB) protocol [5] to seek an uncongested channel, i.e., to prevent packet loss due to collisions, and to reduce the end-to-end delay of the information transfer. Finally, our proposal employs a mechanism of Store-Carry-Forward (SCF) to mitigate the intermittently connected network problem presented on streets or roads that have low-density traffic conditions in which the number of vehicles is not enough to disseminate data messages using multi-hop communication.

Our contributions in this article can be summarized as follows:

- Review two mathematical models of game-theoretical.
- Adapt two game-theoretical models to VANETs.
- Propose an Adaptive Distributed Dissemination (ADD) protocol to perform data dissemination through two game-theoretical mechanisms.
- Adapt the beacon rate according to the requirements of ATB protocol [5] and influences of urban scenarios without a backhaul communication infrastructure.
- Compare our proposal against others protocols with different traffic densities in terms of packet delivery ratio (PLR), average packet delay (APD), broadcast overhead (BO) and number of collision packets (NCP).
- Evaluate our proposal for video warning message dissemination in terms of frame delivery ratio (FDR) and average peak signal to noise ratio (PSNR).

The remainder of this paper is organized as follows: Section 2 discusses the relevant related work in this area. Section 3 explains the game-theoretical formulation. Section 4 details the game formulation used in vehicular networks. Section 5 presents the ADD protocol. Afterwards, Section 6 discusses the performance evaluation and includes results of our analysis. Finally, some conclusions and future work are pointed out in Section 7.

## 2. Related Work

In this section, we will explore some of the most important works that have been proposed in the literature. Data dissemination in general, is a well-studied topic in VANETs. Among the main solutions that focus on urban scenarios are UV-CAST [6] and AMD [7]. UV-CAST protocol uses digital map information to verify if the vehicle is at an intersection or not. This condition creates additional directional message broadcasts to other road directions. Additionally, UV-CAST can assign to more than one vehicle the responsibility for opportunistic forwarding (SCF), so vehicles can forward the message more than once. AMD (Adaptive Multi-directional data Dissemination) protocol disseminates the message to multiple addresses, which are adjusted adaptively according to the local map and the GPS information. UV-CAST [6] and AMD [7] handle a similar scheme that combines broadcast suppression and Store-Carry-Forward, i.e., these proposals tackle the broadcast storm and the disconnected network problems simultaneously. According to the good results, this combination is an important basis for the development of a dissemination protocol for road safety applications. The region of interest (ROI) for disseminating emergency messages in urban area was studied in [8]. They proposed a protocol called RCP (Road Casting Protocol), designed to send emergency messages to a group of vehicles identified by the road segment on which they are located. Each receiver of an emergency message decides to forward the message based on the incident and the receiver’s location relative to a point called Critical Junction (CJ). This point is an intersection beyond which a vehicle cannot avoid the blocked road segment. To select a vehicle to forward the message, the protocol is based on two factors: distance and link quality. Some dissemination protocols also adapt their message dissemination strategy to the vehicles’ density. For instance, in [9], authors designed two approaches for dissemination: NFS (Neighbor Store and Forward), a protocol for scenarios with low traffic density; and NJL (Nearest Junction Located), a scheme for vehicular scenarios with high density. Both protocols maintain a list of neighbors which is constructed by exchanging beacon messages. NSL is designed to relay the message only if the vehicle is the closest to any intersection. NSF is based on an opportunistic forwarding mechanism.

On the other hand, we have analyzed some video warning message dissemination proposals. A video warning message provides an accurate overview of the emergency situation. However, the reliable dissemination of video content using multi-hop broadcast techniques also suffers from the broadcast storm problem and the interference from the existing periodic single-hop beacon messages. The main purpose of most articles on video transmission is entertainment on highways, so the video is streamed from Road Side Units (RSU) to the vehicular network. However, we focus on urban scenarios where the vehicles’ traffic is relatively dense and the communications are more exposed to interferences and fading phenomena. In this sense, authors in [10] proposed a rebroadcasters selection mechanism for video streaming over VANET in urban scenarios. This solution selects a subset of vehicles to rebroadcast the content, based on their strategic location in the network and their capacity to reach a maximum amount of vehicles in a minimum number of hops. The recent adoption of High Efficiency Video Coding (HEVC) known as H.265 standard [11] provides many opportunities for new multimedia services in VANETs. For instance, one of the works where the use of H.265 codec was evaluated in VANET environments was presented in [12]. In this work, authors combined different flooding techniques and different video codecs to assess the effectiveness of long–distance real–time video streaming. According to the results presented by the authors, H.265 shows to perform better than the H.264 codec, being more robust under high packet loss levels.

Although several published works addressed the problems of video content delivery in VANETs, few works have been reported on real-world measurements of visual quality for video. One of them is presented in [13] where authors propose a system called the See-Through System (STS) that relies on VANET and video-streaming technology. The STS allows the overtaking vehicle to have the visual perspective of the road of the preceding vehicle, enhancing the driver’s visual perception of vehicles traveling in the opposite direction lane. Authors also implemented a realistic driving simulator where the usability of the system is further evaluated.

During the past decade, game theory experienced a strong surge of interest in the area of wireless communications. Wireless networks have evolved enormously during this time, making game theory especially relevant in their analysis and design. In our work, we propose a decentralized stochastic and zero-infrastructure support dissemination protocol for applications in urban areas. The key difference between our proposal and others related with game theory (e.g., [14,15,16]) is that the probability of forwarding is strictly dependent on the asymmetric cost–utility ratio and on the size of the group, whereas, in other proposals, the probability of forwarding depends only on the symmetric cost–utility ratio. For instance, an optimized utility function based on distance and mobility was proposed in [14] for enhancing data dissemination in VANETs. In [15], authors propose a data dissemination protocol for VANETs that distributes data utility fairly over vehicles while adaptively controlling the network load. In this case, the protocol relies only on local knowledge to achieve fairness with concepts of Nash Bargaining from game theory. Among all the protocols proposed in literature, the most similar to the one presented in this work was proposed in [16] as a technique to mitigate the broadcast storm problem through a game-theoretical mechanism. With this mechanism, the forwarding probability is a symmetrical game where all players computes an identical cost–benefit ratio. However, few studies can be found that address the asymmetric information as the basis for decision making; that is, all players compute a forwarding probability under different costs or utility. Compared with other solutions, our scheme is a cross layer dissemination protocol capable to achieve higher reception rate and higher video quality over the variant vehicular density using asymmetric information. In this work we do not propose new factors to be considered in dissemination, since there already are a lot of good proposals in the literature (e.g., [17]). The goal of this work is to propose a new way to consider those factors with major impact, using game-theoretical algorithms that have been adapted to the problem of smart dissemination in urban scenarios. In our present work, we propose a completely new way to consider key factors with impact (specifically, distance factor, link quality factor and available bandwidth estimation), using two game-theoretical algorithms that have been adapted to the problem of smart messages dissemination in urban scenarios.

## 3. Game-Theoretical Approaches for Dissemination in VANETs

According to [18], the essence of game theory is the mathematical study of interactions between independent decision-makers (refered as *players* of the game) who can have common interests or conflicting. What the other players do has an impact on each decision-maker, whose benefit or utility not only depends on its decisions but also on the others’ decisions. The problem in interactive situations is that the optimal decision (strategy) is unclear, because no player completely controls the final outcome. This means that the problem must be defined before it can be solved. Game theory is a means of proposing, designing interaction models, studying the conditions under which some outcomes can be reached, and designing good strategies [19]. In this section, we summarize two game-theoretical models proposed to mitigate the broadcast storm problem. The two models consider vehicles as intelligent entities capable of observing a structured environment and of deciding whether or not to forward packets. In the following, we present two new approaches for smart dissemination in urban VANETs based on two well-known games: the Asymmetric Volunteer’s Dilemma Game [3,4,20] and the Forwarding Game [21].

### 3.1. First Game-Theoretical Algorithm Designed for Dissemination in VANETs: Asymmetric Volunteer’s Dilemma

The Volunteer’s Dilemma Game modeled by Diekmann [20] is a game composed of *N* players in which each individual prefers to avoid the cost of volunteering and exploit the benefit of the collective goods produced by others, although someone must volunteer. Defection is the dominant strategy from the perspective of individual rationality. Nevertheless, it becomes collectively irrational if all players in the group choose to free ride. If there is no volunteer in the group, all lose. Everyone can be better off by playing the dominated strategy which explains the existence of a dilemma. The basic game model is defined as:

(1) G={N,S,K,U},N⩾2,

where *N* is the number of players, S={Cooperation,Defection} is the strategy set that dictates player responses to stimuli in the external environment, K>0 is the cost of volunteering (Cooperation), and *U* is the benefit earned when at least one player volunteers.

In this type of game, there are *N* asymmetric equilibria in pure strategies, i.e., cooperation (*C*) and defection (*D*), in which exactly one player, the volunteer, contributes [20]. They are usually attainable with coordination amongst players. With *N* players, there is a equilibrium point that is symmetric if mixed strategies are introduced. Letting $\beta_{i}$ be the probability of player i’s D-choice (not volunteering), the expected utility is:

(2) $$E_{i}=\overset{\mathrm{Defect}\;(D)}{\overset{︷}{\beta_{i}\cdot U_{i}\cdot \left(1-\prod_{j\neq i}^{N}\beta_{j}\right)}}+\underset{\mathrm{Collaborate}\;(C)}{\underset{︸}{\left(1-\beta_{i}\right)\cdot \left(U-K\right)}}$$

The mixed-strategy equilibrium can be found by taking the derivative with respect to $\beta_{i}$ and letting $\frac{dE_{i}}{d\beta_{i}}=0$ , which gives:

(3) $$\beta_{eq}={\left(\frac{K}{U}\right)}^{\frac{1}{N-1}}$$

A key assumption is strict symmetry in terms of the costs of volunteering (*K*) and benefit (*U*) of all players. Thus, this version of the game is referred to as symmetric volunteer’s dilemma. Conversely, Diekmann presented an analysis of an asymmetric volunteer’s dilemma game [3]. In that version of the game, the author introduced an unequal distribution of cost of volunteering $K_{i}$ and benefit $U_{i}$ earned when at least one player *i* volunteers in a group of size *N* players. If we let strategy $D_{i}$ be played with probability $\beta_{i}$ , the expected utility of player *i* can be expressed as follows:

(4) $$\begin{array}{c} E_{i}=\overset{\mathrm{Defect}\;(D)}{\overset{︷}{\beta_{i}\cdot U_{i}\cdot \left(1-\prod_{j\neq i}^{N}\beta_{j}\right)}}+\underset{\mathrm{Collaborate}\;(C)}{\underset{︸}{\left(1-\beta_{i}\right)\cdot \left(U_{i}-K_{i}\right)}} \end{array}$$

where $\beta_{i}$ is the player, *i*’s probability of defection, $U_{i}$ is the benefit earned by that player when at least one player volunteers, $\beta_{j}$ is the average defection probability of all the other players ( j≠i ), and $K_{i}$ is the cost of volunteering for that player *i*.

The best response function for player *i* can be obtained by maximizing Equation (4), we get the solution of the best response for player *i*:

(5) $$\begin{array}{c} \beta_{i}^{*}=\frac{U_{i}}{K_{i}}\cdot {\left(\prod_{j=1}^{N}\frac{K_{j}}{U_{j}}\right)}^{\frac{1}{N-1}} \end{array}$$

Based on Equation (5), the Nash-equilibrium strategy implies that node i’s defection probability will increase with decreasing the value of $K_{i}$ or increasing the value of $U_{i}$ . All variables presented in this game are defined in Table 1.

The asymmetric volunteer’s dilemma game would be played in a VANET whenever a vehicle receives a broadcast message that must be forwarded. Each receiving vehicle computes its $\beta_{i}$ in equilibrium using Equation (5). Thus, each vehicle could choose in a decentralized way its best strategy. Afterwards, in Section 4, we adapt the asymmetric volunteer’s dilemma game to VANETs.

### 3.2. Second Game-Theoretical Algorithm Designed for Dissemination in VANETs: Forwarding Game

Unlike the volunteer’s dilemma, in the Forwarder Game modeled by Naserian [4], the outcome is the probability that each node forwards the message. Upon receiving the flooding packet, the neighbors of a source node *i* choose the forwarding probability as their strategy according to the following game *G*.

(6) $$G=\{N,S_{i},U_{i}\},\;N⩾2,\;i\in N$$

where *N* is the number of players of the game, $S_{i}$ is defined as the probability that node *i* forwards the received message ( $0<S_{i}\leq 1$ ), and $U_{i}$ is the utility earned when at least one node forwards the received message.

As our main goal is to mitigate the broadcast storm problem and therefore improve the overall performance of the network by eliminating redundant broadcast, we used the utility function $U_{i}$ modeled by Naserian [4] and defined as:

(7) $$U_{i}\left(S_{i},a_{i},Q_{i}\right)=\frac{a_{i}\cdot S_{i}}{Q_{i}}\cdot exp\left(\frac{-S_{i}^{2}}{2\cdot k\cdot a_{i}^{n}\cdot Q_{i}^{m}}\right)$$

where *k*, *m* and *n* are constant values, $a_{i}$ is the availability of node *i*, and $Q_{i}$ is the neighbor action reflection.

We identify the availability of a node $a_{i}$ and the strategy of its neighboring nodes $S_{-i}$ as main metrics that allow node *i* to select a strategy $S_{i}$ that maximizes its utility $U_{i}$ . First, we design the availability $a_{i}$ of a node *i* as a multimetric parameter that measures the amount of resources available for that node. This estimated value is a normalized average ( $0<a_{i}\leq 1$ ) of some relevant parameters in our network such as available bandwidth and node’s position. Next, a node *i* can estimate its neighbors’ participation from the information provided by beacon messages. This estimated parameter is called neighbor action reflection, denoted by $Q_{i}$ and defined as:

(8) $$Q_{i}=1-S_{-i}$$

$Q_{i}$ generates a balance between the probability of participation of node *i* and the average forwarding probability of the neighboring nodes of *i*.

Setting the derivative of Equation (7) equal to zero, we get an expression that allows us to calculate the maximum utility of node *i* as a function of parameters $a_{i}$ and $Q_{i}$ :

(9) $$S_{i}^{*}=\sqrt{k\cdot a_{i}^{n}\cdot Q_{i}^{m}}\leftarrow \mathrm{Strategy}\;\mathrm{that}\;\mathrm{maximizes}\;\mathrm{the}\;\mathrm{utility}\;\mathrm{function}\;\mathrm{of}\;\mathrm{node}\;i$$

In the forwarding game, a node *i* with *N* neighbors can estimate the average forwarding probability $S_{i}$ of the other nodes as:

(10) $$S_{-i}^{*}=\sum_{\begin{array}{c} j=1\\ j\neq i \end{array}}^{N}\frac{S_{j}}{N-1}$$

Equilibrium is a term used in game theory to describe a point where each player’s strategy is optimal given the strategies of all other players. In this sense, every node can find its best strategy to play the game replacing Equations (8) and (10) in Equation (9), so we have:

(11) $$\begin{array}{c} \begin{array}{cc} {S_{i}}^{*} & =\sqrt{k\cdot a_{i}^{n}}\cdot {\left(1-\sum_{\begin{array}{c} j=1\\ j\neq i \end{array}}^{N}\frac{S_{j}}{N-1}\right)}^{\frac{m}{2}} \end{array} \end{array}$$

We design Equation (11) with k=4 , n=3 and m=2 since these are optimal values. In addition, we rename the availability factor $\alpha_{i}=\sqrt{4\cdot a_{i}^{3}}$ . Finally, we obtain an expression for the best forwarding probability of node *i*:

(12) $$\begin{array}{c} \begin{array}{cc} S_{i}^{*} & =\alpha_{i}\cdot \left(1-\sum_{\begin{array}{c} j=1\\ j\neq i \end{array}}^{N}\frac{S_{j}}{N-1}\right) \end{array}\; \end{array}$$

Equation (12) consists of *N* linear equations for each player *i*. Thus, every node can solve the system of equations and find its best strategy to play the game. We assume that each node *i* knows the value of its availability factor $\alpha_{i}$ and number of neighboring nodes of node *i*, $N_{i}$ . In Section 4 we will see how nodes compute their $\alpha_{i}$ in the designed game to improve dissemination in VANETs.


(13) $$\left[\begin{array}{c} S_{1}\\ S_{2}\\ S_{3}\\ \vdots \\ S_{N} \end{array}\right]\left[\begin{array}{cccccc} 1 & \frac{\alpha_{1}}{N-1} & \frac{\alpha_{1}}{N-1} & \cdots & \frac{\alpha_{1}}{N-1} & \alpha_{1}\\ \frac{\alpha_{2}}{N-1} & 1 & \frac{\alpha_{2}}{N-1} & \cdots & \frac{\alpha_{2}}{N-1} & \alpha_{2}\\ \frac{\alpha_{3}}{N-1} & \frac{\alpha_{3}}{N-1} & 1 & \cdots & \frac{\alpha_{3}}{N-1} & \alpha_{3}\\ \vdots & \vdots & \vdots & \ddots & \vdots & \vdots \\ \frac{\alpha_{N}}{N-1} & \frac{\alpha_{N}}{N-1} & \frac{\alpha_{N}}{N-1} & \cdots & 1 & \alpha_{N} \end{array}\right]$$

Once the results of the system of equations have been found, each node *i* can calculate its best forwarding probability $S_{i}$ . All variables presented in this game are defined in Table 2. In Section 4.2, we will see how to use the designed game in VANETs, and specifically we will design the availability parameter $a_{i}$ in the forwarding node selection process.

## 4. Adapting Both Game-Theoretical Models to Vehicular Ad-Hoc Networks

As we have seen in the previous section, the purpose of Game Theory is to model interactions between players, to define different types of possible outcome, to predict the solution of a game under given information and behavior assumptions, and to design strategies to reach the outcomes. When an emergency message is received by a vehicle, the message should be re-broadcasted by that node. Nonetheless, in the shared wireless medium, unnecessary broadcasts degrade the performance of the network, which is known as broadcast storm problem. In our approach, neighbors of the source node play a game-theoretical algorithm upon receiving the emergency message, and they choose a forwarding probability as their strategy. The outcome of the game is the probability that each node forwards the emergency message.

### 4.1. Design of the Utility Function for the Asymmetric Volunteer’s Dilemma

Using the framework of the volunteer’s dilemma (VoDi) defined in Section 3, we now formulate the VoDi to model warning messages dissemination in VANETs. We consider the special case of an asymmetric volunteer’s dilemma with increasing benefit earned by vehicle *i* when at least one player volunteers ( $U_{i}$ ) and strictly constant costs ( $K_{i}$ ), i.e., $U_{1}>U_{2}>\cdots >U_{N}$ where $U_{i}>K_{i}>0$ . The costs $K_{i}$ have been fixed to 1. This is not a limitation of the analysis. Quite the opposite, it is a grade of freedom of the game that could be used to extend the model based on the benefits earned by vehicle *i* when at least one player volunteers.

Efficient delivery of messages in a VANET depends critically on the set of intermediate nodes which act as forwarding nodes. The behavior of the vehicle that received the message affects positively or negatively the behavior of other vehicles, depending on whether there was a choice of forwarding the message or not. We define several core strategies combined as an integral scheme to enhance the performance and the reliability of the warning message broadcast. Our proposal of utility function includes information about its local neighborhood and significant cross-layer information. Thus, the utility function for node *i* is given by:

(14) $$U_{i}\left(Df_{i},LQf_{i}\right)=10^{\left(10\;-\;\left(\alpha_{1}\cdot Df_{i}\;+\;\alpha_{2}\cdot LQf_{i}\right)\right)}$$

which is composed by two parameters related to information provided by vehicle *i*: the position of the vehicle in the network ( $Df_{i}$ ) and an estimation of the congestion of the communication channel ( $LQf_{i}$ ). ADD calculates the utility $U_{i}$ , which is later used to compute its $\beta_{i}$ in equilibrium. Higher values of $Df_{i}$ and $LQf_{i}$ represent a better position of the vehicle and a better channel conditions, respectively (in the range [0,1] ). Powers of base ten to make the relationship sensitive enough to environmental conditions. The weights $\alpha_{1}=6$ and $\alpha_{2}=4$ have been obtained through extensive simulations and under different traffic conditions, showing best results with those values. Nevertheless, we plan to design a dynamic scheme to update the weights of the multimetric score to calculate the utility function $U_{i}$ for node *i* as pointed out in Section 7. We design the parameters of the utility function proposed in Equation (14) as follows:

**Distance factor** $\left(\alpha_{1},{\mathrm{Df}}_{i}\right)$ : The distance factor is adapted to the information provided by the neighbor discovery process as well as to the information provided by the own GPS device. Thanks to the information gathered by beacon messages, the distance source–receiver $D_{\mathrm{sr}}$ between transmitter and receiver can be calculated. On the other hand, the information provided by GPS allows us to know the receptor-intersection distance $D_{\mathrm{rint}}$ to the next nearest intersection. If the receiving vehicle is not located over an intersection, the distance factor ${\mathrm{Df}}_{i}$ is calculated as the ratio between $D_{\mathrm{sr}}$ and the transmission range $R_{\max}$ . With this, the farthest vehicle from the sender is assigned the highest distance factor. On the other hand, if the receiving vehicle is located over an intersection, the distance factor ${\mathrm{Df}}_{i}$ is calculated as a decreasing function of the distance $D_{\mathrm{rint}}$ . This way, the lower $D_{\mathrm{rint}}$ , the higher its ${\mathrm{Df}}_{i}$ which means that the vehicle is a good candidate to broadcast the message. The distance factor is summarized in Equations (15a) and (15b).

Dfi=(15a)DsrRmax,ifDrint>Rmax(15b)1−DrintDrint+1,otherwise

where $D_{\mathrm{sr}}$ is the relative distance between source s and receptor r vehicles, $D_{r\mathrm{int}}$ is the relative distance between vehicle r and the next nearest intersection, and $R_{\max}$ is the transmission range.

**Figure 1:**
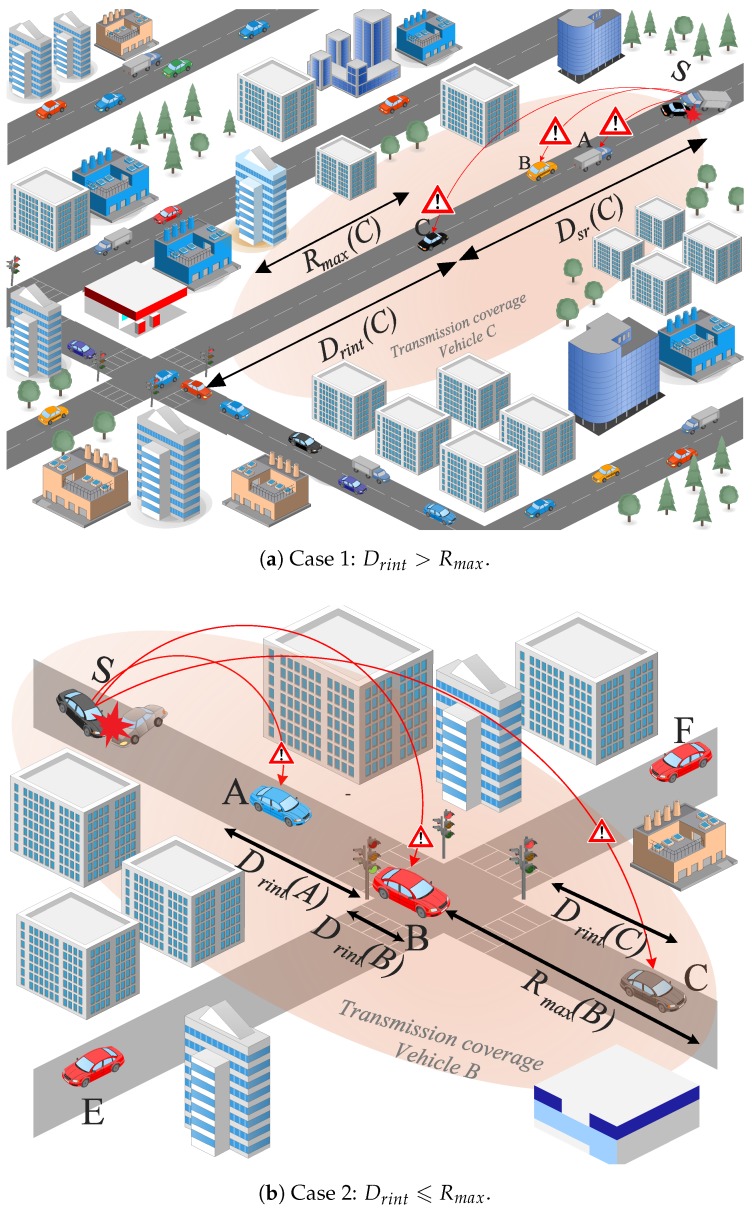
Distance factor $Df_{i}$ .

As it can be seen in Figure 1a, vehicles A, B, and C do not have intersections within their transmission range $R_{max}$ . In this case, the vehicles receiving the message compute the distance factor according to Equation (15a). Thus, vehicle C which is farther from the sending vehicle S will be assigned the highest distance factor without taking into account its distance to the intersection, according to Equation (15a). On the other hand, Figure 1b presents the scenario when the vehicles receiving the message have intersections within their transmission range. In this case, vehicles *A*, *B*, and *C* compute the distance factor according to Equation (15b). Hence, vehicle B that is crossing the intersection is assigned the highest distance factor.

**Link Quality factor** $\left(\alpha_{2},LQf_{i}\right)$ : The Link Quality factor $LQf_{i}$ is a metric designed to indicate the signal quality $\left(sq_{i}\right)$ , the channel quality $\left(cq_{i}\right)$ and the collision probability $\left(cp_{i}\right)$ , which are explained below. The vehicle *i* receiving the message attains those parameters from its physical layer and MAC layer following a cross-layer design. The Link Quality factor is calculated as follows:

(16) $$LQf_{i}=0.5\cdot sq_{i}+0.5\cdot cq_{i}\cdot \left(1-cp_{i}\right)$$

This way we equally add the effects of the signal quality and the channel being free of collision. The signal quality $sq_{i}$ aims at ensuring the integrity of the received message in vehicle *i*, and it is calculated as follows:

(17) $$sq_{i}=\left\{\begin{array}{cc} \max(0,Sf_{iRSS}\cdot \left(1-\frac{1}{SNR_{i}}\right)\cdot \left(1-V_{i}\right)) & ,\;\mathrm{if}\;SNR_{i}>0\\ Sf_{iRSS} & ,\;\mathrm{otherwise} \end{array}\right.$$

where $V_{i}$ is the ratio between the relative velocity (between the transmitter and the receiver of the warning message) and the maximum allowed velocity in the considered urban scenario. The velocity of the vehicle *i* is an influencing parameter in signal quality because the communication link with a vehicle moving at a very high relative speed is less stable than with a vehicle moving at the lower relative speed. Therefore, vehicles moving at high relative speed will obtain low values of $sq_{i}$ . $SNR_{i}$ is the ratio between the signal power and the noise intensity of the *i*-th receiver. Note that those vehicles which are farther from the sender will have lower $SNR_{i}$ . $Sf_{iRSS}$ is the received signal strength in vehicle *i* bounded by 1 and it is defined using the following equation:

(18) $$Sf_{iRSS}=\left\{\begin{array}{cc} \min(1,\frac{RSS_{i}-RSS_{\mathrm{th}}}{RSS_{\max}-RSS_{\mathrm{th}}}) & ,\;\mathrm{if}\;RSS_{i}\geq {\mathrm{RSS}}_{\mathrm{th}}\\ 0 & ,\;\mathrm{otherwise} \end{array}\right.$$

where $RSS_{i}$ is the received signal strength, ${\mathrm{RSS}}_{\mathrm{th}}$ is a threshold below which the received signal is considered too weak and ${\mathrm{RSS}}_{\max}$ is the maximum value of the received signal strength.

The channel quality ( $cq_{i}$ ) is defined in Equation (19) as an estimation of the state of the channel around the receiving vehicle *i* at the time of reception of the message. The ( $cq_{i}$ ) is calculated using the Number of Successful Transmissions (nst) and the Number of Overall Transmissions (not) in a window time. The Number of Successful Transmissions (nst) is an internal statistical parameter that represents packets that were successfully processed in the medium access control (MAC) layer, that is to say, packets that do not suffer bit errors or collisions. A large number of packets lost over the last time window is an indicator that the quality of the channel is poor.


(19) $$cq_{i}=\left\{\begin{array}{cc} \frac{nst_{i}}{not_{i}} & ,\;\mathrm{if}\;not_{i}>0\\ 0 & ,\;\mathrm{otherwise} \end{array}\right.$$

The collision probability (cp) is defined in Equation (20) as an estimation of the likelihood of a collision occurrence if the message is forwarded by the receiver vehicle *i*. It is calculated using the channel occupancy time (cot) and a fixed window time (wt) in which the channel is observed. The channel occupancy time is computed by the MAC layer, which gives us the accumulated time that the channel was busy at the time of the query *t*.


(20) $${\mathrm{cp}}_{i}\left(t\right)=\frac{\cot_{i}\left(t\right)}{\mathrm{wt}(t)}$$

Once the utility function $U_{i}$ has been computed, each vehicle inserts its $K_{i}/U_{i}$ into the beacon message. As the beacon messages are received by each vehicle, they can calculate the factor $\left(\prod_{j=1}^{N}\frac{K_{j}}{U_{j}}\right)$ . When a vehicle creates an emergency message, it inserts the game information into the message, i.e., the factor ${\left(\prod_{j=1}^{N}\frac{K_{j}}{U_{j}}\right)}^{\frac{1}{N-1}}$ . When a vehicle receives an emergency message, it multiplies its current factor $\frac{U_{i}}{K_{i}}$ by the factor included in the emergency message. In this instant, the vehicle *i* is able to calculate its $\beta_{i}^{*}$ in equilibrium according Equation (5). Thus, the asymmetric volunteer’s dilemma game is played whenever vehicles receive a broadcast message and they choose in a decentralized way its best strategy.

### 4.2. Design of the Availability Function in the Forwarding Game

We have designed the availability function of a vehicle as a parameter $a_{i}$ ( $0<a_{i}\leq 1$ ) that measures the amount of available resources in the node *i* that make more efficient the process of disseminating emergency messages. This estimated value is a normalized average of two parameters: estimated available bandwidth and position of the node in the network.

Due to a possible large number of vehicles sharing the wireless medium, it is unclear whether the channel capacity is sufficient to support the data generated by beacon messages and at the same time leaving enough available bandwidth for supporting other applications. For the specific case of emergency video dissemination, the overall capacity of the channel can affect the effectiveness of emergency dissemination schemes if the density of potential forwarders is high. Definitely, available bandwidth estimation is a key component for quality of service (QoS) in VANETs. We have considered the ABE proposal presented in [22] as one solution to estimate the available bandwidth in the link formed by two sender-receptor vehicles. ${\mathrm{ABE}}_{\left(s,r\right)}$ aims to provide an accurate estimation of the available bandwidth in the link formed between two neighbor nodes *s* and *r*, which can be estimated by the following equation:

(21) $$ABE_{\left(s,r\right)}=\left(1-K_{ABE}\right)\cdot \left(1-cp\right)\cdot T_{s}\cdot T_{r}\cdot C_{ABE}$$

where $K_{ABE}$ is the proportion of bandwidth used by the back-off scheme which is estimated with Equation (22). cp is the collision probability measured on the received Hello packets and it is computed with Equation (20). $T_{s}$ is the idle time period at the sender side and $T_{r}$ is the idle time period at the receiver node, and *C* is the maximum medium capacity on link ( s,r ).

(22) $$K_{\mathrm{ABE}}=\frac{\mathrm{DIFS}+\bar{\mathrm{backoff}}}{T_{m}}$$

where $T_{m}$ (in second) is the time elapsed between the emission of two consecutive frames, DIFS (Distributed Coordination Function Interframe Space) is a fixed interval and $\bar{backoff}$ is the mean backoff used to transmit a single frame.

On the other hand, we have taken into account the position of the receiver node in the network to design a measure of the amount of available resources $a_{i}$ for vehicle *i*. As mentioned previously, vehicles located in intersections typically have better network connectivity than non-intersection vehicles. In addition, vehicles whose location is farthest from the source are potential forwarders in the process of message dissemination. In addition, position of the vehicles in the road-map has a large impact on the efficiency of dissemination due to the effect of buildings. With all these considerations, the position of the node is evaluated similarly to the distance factor ${\mathrm{Df}}_{i}$ presented in Equation (15).

To compute the availability $a_{i}$ , we first divide ABE by the link capacity $C_{ABE}$ , obtaining a normalized available bandwidth metric. A high value of ABE means a high available bandwidth in the link formed by both vehicles *s* and *r*. Next, we also consider the distance factor ${\mathrm{Df}}_{i}$ as a function of the distance between the sender and the potential next forwarder *i*, and the next nearest intersection $D_{r\mathrm{int}}$ . Equation (23) presents the availability function $a_{i}$ of the potential forwarder.

(23) $$a_{i}=\frac{ABE_{i(s,r)}}{C_{ABE}}\cdot \gamma +{Df}_{i}\cdot \left(1-\gamma \right)$$

where γ=0.5 is a weight to average both metrics.

**Figure 2:**
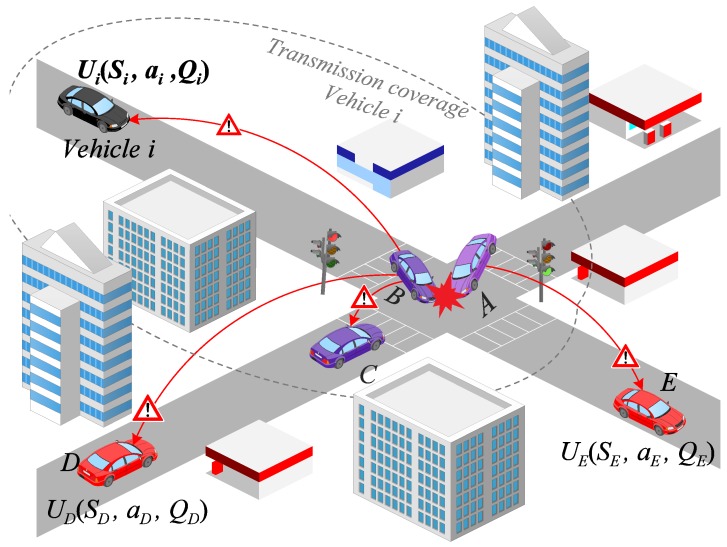
Forwarding Game in an urban scenario.

Figure 2 shows the variables present in the Forwarding Game where each vehicle must select a strategy that maximizes its utility. Below we detail the Forwarding Game for the black vehicle *i*. Each vehicle inserts its availability factor $\alpha_{i}=\sqrt{4\cdot a_{i}^{3}}$ into the beacon message. As the beacon messages are received by each vehicle, black vehicle *i* can storage the availability factors $\alpha_{A},\alpha_{B}$ and $\alpha_{C}$ of its neighbors A,B and *C*, respectively. When black vehicle *i* receives an emergency message, it should compute the parameters availability factor $\alpha_{i}$ and neighbor action reflection $Q_{i}=1-S{}_{-i}$ , as it is detailed in Section 3.2. While the availability of vehicle *i* depends exclusively on information of itself, $Q_{i}$ is estimated based on the information of the strategy of its neighbors (purple vehicles *A*, *B* and *C*), i.e., $S_{-i}=\sum_{\begin{array}{c} j=1\\ j\neq i \end{array}}^{N}\frac{S_{j}}{N-1})$ allows us to estimate the parameter $Q_{i}$ . Note that red vehicles *D* and *E* are outside the transmission coverage of vehicle *i*, so vehicle *i* does not receive their beacons. Finally, vehicle *i* selects its best strategy $S_{i}$ to obtain a maximum utility $U_{i}$ according Equation (12). Thus, the forwarding game is played whenever vehicles receive a broadcast message and they choose in a decentralized way their best strategy to broadcast the message or not.

## 5. Adaptive Distributed Dissemination Protocol Description

This section describes the proposed protocol called Adaptive Distributed Dissemination (ADD). The main goal of the ADD mechanism is disseminate warning messages to all vehicles inside the region of interest (ROI) independently of the road traffic condition. Therefore, our proposal must be able to tackle the broadcast storm and intermittently connected network problems.

We assume that each vehicle is equipped with an GPS device to obtain its geographical location in current time. A preloaded digital map provides information about roads and intersections. Besides, we assume that vehicles periodically exchange their physical location, moving velocity and moving direction enclosed in their periodic beacon messages. Finally, vehicles are assumed to be equipped with IEEE 802.11p wireless technology and computation capabilities.

### 5.1. Adaptive Distributed Dissemination (ADD) Scheme

ADD is a data dissemination protocol based on a periodic beacon-based approach, so-called Basic Safety Message (BSM) [23]. ADD is located on top of UDP (User Datagram Protocol) and WSMP (WAVE Short Message Protocol) [24]. The WSMP protocol is meant to handle safety messages, whereas non-safety messages can be sent with either WSMP or UDP. ADD includes five main modules: neighbor discovery, relaying node selection, store-carry-cooperative forward, adaptive beaconing and video quality strategy, which are described in the following.

#### 5.1.1. Neighbor Discovery

The neighbor discovery mechanism in ADD keeps the knowledge of the local topology by monitoring periodic beacon updates received from one-hop neighbors. Each vehicle periodically announces its status to all its one-hop neighbors by broadcasting a beacon packet. These packets carry the current location of the node which is acquired from the GPS, moving velocity and moving direction. In addition, each beacon contains the IDs of the warning messages that have been received and are being carried by the vehicle. Note that incorporating the IDs of the received data messages into the beacons works as an implicit acknowledgment mechanism. Therefore, when a vehicle receives a beacon from a neighbor, it is able to verify if it has any warning message that has not been received by this neighbor and then forward it accordingly. Each vehicle sets up and dynamically updates a neighbors table that contains identification, mobility information of all one-hop surrounding vehicles and IDs of received warning messages.

#### 5.1.2. Relaying Node Selection in ADD

Relaying is the task of assigning the duty of forwarding a message to a specific node or nodes that satisfy some criteria. Our approach ADD is able to respond to environmental changes, adapting its operation mode to face those frequent topology changes inherent in VANETs. Two types of relaying node selection are presented based on two game-theoretical schemes. The games are played whenever a vehicle receives a broadcast message that must be forwarded. Algorithms 1 and 2 show the *Volunteer’s Dilemma* relaying scheme and the forward game relaying scheme, respectively.

**Algorithm 1** Asymmetric Volunteer’s Dilemma Operation**Require:** Neighbor Table, Warning Message (
warningMsg
)**Ensure:** Select forwarder node
1:**procedure** receiveWarningMessage (*Warning Message)2:    **if** WarningMsgTable.getId = warningMsgId **then**3:        Discard packet4:        **if** rebroadcast timer for warningMsg is scheduled **then**5:           Cancel rebroadcast timer for warningMsg
6:        **end if**7:    **else** 
// Embedding IDs of received warning messages into beacons8:        Add warningMsgId in subsequent beacons 
// Best response for vehicle *i*, see Equation (5)9:        
$\beta_{i}\leftarrow \frac{U_{i}}{K_{i}}\cdot {\left(\prod_{j=1}^{N}\frac{K_{j}}{U_{j}}\right)}^{\frac{1}{N-1}}$
10:        
rnd←uniform(0,1)
11:        **if** $rnd<(1-\beta_{i})$ **then**12:           Node selected for forwarding13:           Forward packet14:           Storage in temporary buffer15:        **else**16:           Wait for a Backoff17:           **if** packet was not forwarded by another vehicle **then**18:               Forward packet19:               Storage in main buffer20:           **end if**21:        **end if**22:    **end if**23:
**end procedure**



The first game-theoretical forwarding scheme uses the *Volunteer’s Dilemma* (see Section 1) to select the relaying node. According to Algorithm 1, the Relaying node selection module executes the procedure $receiveWarningMessage{(}^{*}WarningMessage)$ (line 1). If a warning message with identifier warningMsgId was previously received (line 2), the message is accounted and discarded (line 3). In addition, if the vehicle receives a duplicate warning message while it is scheduled to rebroadcast warningMsg , then it cancels the rebroadcast (lines 12 and 13), thus avoiding a possible redundant retransmission. Otherwise, if a warning message is new, vehicle will insert the identifier warningMsgId into subsequent beacons, until warning message expires (line 8). After that, the vehicle computes the probability of defection $\beta_{i}^{*}$ using Equation (5), as a function of the benefit earned by that player when at least one player volunteers ( $U_{i}$ ), the average defection probability of all the other players ( $\beta_{j}$ ), the cost of volunteering for that player *i* ( $K_{i}$ ) and the average cost of volunteering of all the other players *j* (line 9). Once calculated $\beta_{i}^{*}$ , the receiveWarningMessage() procedure provides a random number rnd() to be compared with the probability to volunteer (i.e., to forward the packet) $1-\beta_{i}^{*}$ (line 11). If $rnd\left(\right)<\left(1-\beta_{i}^{*}\right)$ then vehicle *i* is selected as a forwarding node and rebroadcasts this warning message (lines 12 and 13). Otherwise, if the vehicle does not forward the message, a timeout WT is assigned to the node (line 16). Note that in Equation (24), the vehicle farthest from the transmitting node will have assigned the shortest WT period. After the waiting time expiration, the forwarder vehicle rebroadcasts the message only if the node has not received a copy of the message (lines 17 and 18) and stores data packet in the main buffer (line 19).

**Algorithm 2** Forwarding Game Operation**Require:** Neighbor Table, Warning Message (
warningMsg
)**Ensure:** Select forwarder node
1:**procedure** receiveWarningMessage(*Warning Message)2:    **if** WarningMsgTable.getId = warningMsgId **then**3:        Discard packet4:        **if** rebroadcast timer for wm is scheduled **then**5:           Cancel rebroadcast timer for Warning Message wm
6:        **end if**7:    **else** 
// Embedding IDs of received warning messages into beacons8:        Add warningMsgId in subsequent beacons 
// Best response for vehicle *i*, see Equation (12)   9:        
$S_{i}^{*}\leftarrow \sqrt{k\cdot a_{i}^{n}}\cdot \left(1-\sum_{\begin{array}{c} j=1\\ j\neq i \end{array}}^{N}\frac{S_{j}}{N-1}\right)$
   10:        
rnd←uniform(0,1)
11:        **if** $rnd<S_{i}^{*}$ **then**12:           Node selected for forwarding13:           Forward packet14:           Storage in temporary buffer15:        **else**16:           Wait for a Backoff17:           **if** packet was not forwarded by another vehicle **then**18:               Forward packet19:               Storage in main buffer20:           **end if**21:        **end if**22:    **end if**23:
**end procedure**



The second game-theoretical forwarding scheme designed uses the *Forwarding Game*, explained in Section 2, to select the relay node or nodes. The proposed mechanism is further described in Algorithm 2. The general scheme of this game is similar to the *Volunteer’s Dilemma*. The main difference is that the forwarding probability of a source node *i* is now calculated as a function of the availability of node *i* (line 9). Any of its neighbors that hears a duplicate rebroadcast, with regard to the recently received message, will cancel its message-rebroadcast process.

According to both schemes, each forwarder candidate adjusts its own waiting time WT that is inversely proportional to the distance from itself to the previous forwarder vehicle, as shown in Equation (24).

(24) $$WT=0.005+(SLOT\_TIME\cdot \left(R_{max}-D_{tc}\right))$$

where SLOT_TIME represents a time slot, $R_{max}$ represents the maximum transmission range, and $D_{tc}$ is the distance between the transmitter and the forwarding potential candidate vehicle. Since forwarding candidates are neighbors $D_{tc}⩽R_{max}$ , a vehicle in the edge of the coverage radius will wait a WTmin , while a vehicle close to the sending one will have to wait WTmax . This gives priority to the most distant vehicle in the coverage area to broadcast the message.


(25) $$WT_{2}=0.005+\left(SLOT\_TIME\cdot D_{tc}\right)$$

### 5.2. Store-Carry-Cooperative Forwarding (SCCF)

In this section, we present the operation of a store-carry-forward (SCF) based algorithm that we call store-carry-cooperative forwarding (SCCF). SCF is a conventional data forwarding mechanism in vehicular ad-hoc networks proposed in several works [25,26]. The main idea is that vehicles keep the copies of messages and replicates whenever there is a contact opportunity. Taking advantage of vehicle mobility, relay vehicles are expected to have contact with new neighbors and deliver the message. This mechanism is robust to intermittent network connectivity and can guarantee data delivery. In our proposal, a vehicle forwards messages only when it finds uninformed vehicles. SCCF presents two types of data buffers to store message: main buffer and temporary buffer.

- Temporary buffer: It stores copies of message which are being broadcasted but waiting for duplicated to ensure the successful reception. After the vehicle overhears the duplicated packets from the forwarding vehicle, the corresponding message copy will be deleted from the temporary buffer. Otherwise, the vehicle will recover the message from the secondary buffer and store it in the main buffer after timeout $\delta_{t}$ .
- Main buffer: It stores messages when a vehicle cannot find neighbors within its transmission range.

When the network is partitioned (sparse road traffic), vehicles use their mobility capabilities to carry the stored messages to different parts of the ROI. Furthermore, vehicles must be able to determine if a new neighbor has already received a warning message or not. For this, beacons are used as an implicit acknowledgement mechanism. Algorithm 3 shows how our proposed solution delivers warning messages even when the network is intermittently connected. When a vehicle receives a beacon message $b_{j}$ from a neighbor *j*, it verifies whether there is a warning message that has not been acknowledge by *j* in $b_{j}$ (lines 1–6). For that, the vehicle searches into its main buffer and compares their IDs with the IDs contained in the beacon message $b_{j}$ . If the vehicle finds any message warningMsg that has not been acknowledged, then it calculates a waiting delay $WT_{2}$ to rebroadcast warningMsg . This delay will depend on Equation (25). In this case, vehicles closer to the uninformed neighbor receives a lower waiting time than vehicles farther away. Then, the vehicle schedules to rebroadcast warningMsg with delay $WT_{2}$ (lines 2–4). As in the relaying node selection algorithms, if vehicle receives a duplicate message, it cancels the rebroadcast (lines 7–11), thus avoiding a possible redundant retransmission. However, when the waiting delay $WT_{2}$ expires and the vehicle has not received any duplicate, then it rebroadcasts warningMsg (lines 12 and 14).

**Algorithm 3** Store-Carry-Cooperative Forward Operation**Require:** Beacon Message (*b*), Main buffer,**Ensure:** Hold received data messages and replicates whenever find non-informed neighbors
 
// Check if there is a warning message that has not been acknowledge by neighbor *j* in Beacon *b*.1:**function** **CompareMainBuffer_IDsMsgsBeacon**(**IDsMsgsBeacon,MainBuffer**)2:    **if** message warningMsg is not acknowledged in *b* **then** 
// Calculate a waiting delay $WT_{2}$ to rebroadcast warningMsg . See Equation (25)3:        
$WT_{2}\to$ Waiting Delay4:        Schedule rebroadcast timer for found message warningMsg
5:    **end if**6:
**end function**
 
// A warning message warningMsg is received from neighbor *j*.7:**if** warningMsgId is duplicated **then**8:    **if** rebroadcast timer for warningMsg is scheduled **then**9:        Cancel rebroadcast timer for warningMsg
10:    **end if**11:
**end if**
 
// Rebroadcast timer expires12:**function** **rebroadcastMessage**(*Warning Message)13:    Rebroadcast message warningMsg
14:
**end function**



#### 5.2.1. Adaptive Beaconing

In this section, we summarize the adaptive traffic beacon (ATB) protocol that we have included in our ADD game-theoretical dissemination algorithms. Beaconing is the basic supporting process that enables message dissemination; however, this requires a significant amount of bandwidth. The higher the beaconing frequency, the better the accuracy of neighboring information, but the higher the bandwidth consumption. This means that, if the beacon rate were fixed, channel load could increase too much, especially in scenarios with high vehicle density. To tackle this problem, we have implemented the ATB protocol proposed in [5] that adapts the beacon rate continuously to the current environment circunstances. We compute the beacon interval $\Delta I_{i}$ according to Equation (26):

(26) $$\begin{array}{c} \Delta I_{i}=I_{min}+\left(I_{max}-I_{min}\right)\cdot I_{i} \end{array}$$

where $I_{min}$ and $I_{max}$ represent the minimum and the maximum beacon interval, respectively. The interval parameter $I_{i}$ (in the range [0, 1]) is calculated according to:

(27) $$\begin{array}{c} I_{i}=\left(\left(1-W_{I}\right)\cdot P_{i}^{2}+W_{I}\cdot C_{i}^{2}\right) \end{array}$$

where $P_{i}$ is the beacon message priority and $C_{i}$ represents the current channel condition. The relative impact of those two parameters is configured using an interval weighting factor $W_{I}$ . Smaller values of $P_{i}$ and $C_{i}$ represent a higher priority in the channel access category and a better channel conditions, respectively.

In the following, we briefly introduce the different metrics that the ATB algorithm uses to assess channel conditions and beacon message priority for a given vehicle *i*.


(28) $$P_{i}=\overset{\mathrm{Priority}\;\mathrm{Beacon}}{\overset{︷}{\frac{A_{i}+D_{e_{i}}+D_{r_{i}}}{3}}}$$

(28a) $$A_{i}=\min\left\{{\left(\frac{\mathrm{beacon}\mathrm{message}\mathrm{age}}{I_{max}}\right)}^{2};1\right\}$$

(28b) $$D_{e_{i}}=\min\left\{{\left(\frac{\mathrm{dist}.\mathrm{to}\mathrm{event}/speed_{i}}{I_{max}}\right)}^{2};1\right\}$$

(28c) $$D_{r_{i}}=\max\left\{0;1-\sqrt{\frac{\mathrm{dist}.\mathrm{to}\mathrm{junction}/speed_{i}}{I_{max}}}\right\}$$

(29) $$\begin{array}{c} C_{i}=\overset{\mathrm{Channel}\mathrm{conditions}}{\overset{︷}{\frac{N_{i}+W_{c}\cdot \frac{S_{i}+K_{i}}{2}}{1+W_{c}}}} \end{array}$$

(29a) $$\begin{array}{c} N_{i}=\min\left\{{\left(\frac{\#neighbors_{i}}{\#neighbors_{max}}\right)}^{2};1\right\} \end{array}$$

(29b) $$\begin{array}{c} S_{i}=\max\left\{0;{\left(\frac{SNR_{i}}{SNR_{max}}\right)}^{2}\right\} \end{array}$$

(29c) $$\begin{array}{c} K_{i}=1-\frac{1}{1+\#colisions_{i}} \end{array}$$

Acoording to the U.S. Department of Transportation [27], intersections are potential points of conflict in any roadway system. Therefore, there is a need for heightened caution and attention when vehicles approach intersections. For a final situation-adaptive beaconing scheme, we propose to include the distance to the next intersection as a factor to increase the beacon rate. In this way, when vehicles approach intersections, those vehicles temporarily increase their beacon rate, as presented in Equation (28c). A detailed analysis of all the parameters discussed here can be found in [28].

#### 5.2.2. Video Quality Module in ADD

Disseminating video over a VANET is not an easy task because transmission of video should fulfill timing constraints inherent in the delivery and playback of video content. Besides, supporting video transmission is an attractive feature for a wide variety of services, such as traffic management, infotainment, road emergencies and scientific application. For instance, disseminating a video showing vehicles stuck in a traffic jam could be more effective than receiving a text message for a driver to change the current route. On the other hand, the benefits of smart cities also provide infotainment applications for citizens. In the future, information dissemination base stations could be deployed in shopping mall, museums, theaters and stadiums to send advertisements offering smart services to passing drivers using VANETs.

A key component to efficiently transport video with its stringent playout deadlines and bursty traffic characteristics, is using the most–efficient current encoding format. According to our previous studies [29,30], H.265 allows us to transport higher quality videos with better resolution at the same bit rates of previous generation codecs, reducing overall cost of video delivery while improving on the quality of experience for users. The latest versions of HM (HEVC Test Model) [11] reference software model was used for encoding video sequences with H.265/HEVC. We use two coding parameters: the Constant Rate Factor (CRF) and the encoding mode as a strategy for maintaining high quality video. The video traces were built with the following structure: frame sequence number *n*, cumulative display time $T_{n}$ , frame type (I, P, or B) and frame size $X_{n}$ (in bits).

## 6. Performance Evaluation

We evaluate ADD by means of several simulations to show its feasibility in a realistic urban scenario. We compare its performance with other similar approaches. In the following, we describe the experiments and discuss the results.

### 6.1. Framework

We have employed OMNeT ++ [31] to perform the simulations and SUMO [32] to generate the vehicular movement traces. OMNeT ++ provides a baseline to develop different type of projects which implement models for several network protocols. Two of these projects, INET [33] and VEINS [34], have been put together to provide a vehicular network simulator. SUMO reports to OMNeT++ with the mobility model with vehicles and their current positions at each simulation step using Traci interface. For a more realistic mobility behavior, we defined a scenario including different type of vehicles (car, bus and truck) with an associated probability of occurrence and maximum speed presented in Table 3. All vehicles are moving according to the SUMO standard Krauss driver model. Besides, seeking to dispose a scenario prepared as realistically as possible, we use real maps extracted from OpenStreetMap [35]. Specifically, in this work, we used a map of Berlin, Germany.

### 6.2. Simulation Setup

To carry out the performance of our proposal and compare the results with the other analyzed dissemination schemes, we have prepared each run with a different random scenario that fulfills the requirements of the study. For each point in all figures, we have calculated the average from 10 simulation runs, each with a different seed. This lets us obtain a standard error less than 5% in a 95% confidence interval. The Medium Access Control (MAC) layer is the used in the IEEE 802.11p, using a data rate of 6 Mbit/s, a transmission power of 10 mW, and a receiver sensitivity of −89 dBm. Beacon messages use the Access Category (AC) AC_BE, whereas data traffic uses (AC) AC_data. Internally, ADD calculates the so-called interval parameter *I*, which is later used to adapt the beacon interval in all simulation scenarios. Table 4 contains a summary of the simulation parameters common to all the simulation scenarios evaluated.

### 6.3. Scenario Description

**Figure 3:**
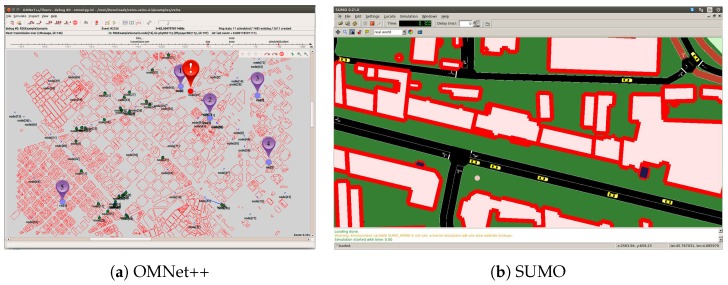
Screenshots of OMNet++ and SUMO simulators’ graphical user interfaces running network and road traffic simulations, respectively. Vehicular network scenario in OMNeT ++ : 2.5 km × 2.5 km urban region in Berlin, Germany (red rectangles = buildings; red circle = crashed vehicle; green circles = warned vehicles; purple circles = RSUs).

We focus on the immediate consequences of an accident in a city road. The crashed vehicle starts to generate and transmit an SOS alert after the collision to warn neighboring vehicles and to alert the appropriate emergency centers (e.g., 112 or 911) as quickly as possible in a distributed way. In a first scenario, we have evaluated the performance of the dissemination of a text message. A vehicle positioned approximately at the center of the network is responsible for generating a single warning message to be disseminated in a time to live (TTL) of 30 s. Additionally, a second scenario is evaluated when the crashed vehicle starts to generate and transmit a short video information of the last 40 s before the crash and the 40 s after the accident in an TTL of 120 s. We have prepared pre-compressed sequences of video and produced trace files with the information needed for the simulation, that is, we prepare the video frames and encapsulate them in packets. We have also included the frame sequence number in order to be able to compare the received decompressed video with the original video sequence. For our evaluation, we have used an *Urban* video stream, which is publicly available at [39]. It is the CIF (Common Intermediate Format) version which contains 2400 frames encoded with H.265/HEVC [11]. Constant Rate Factor (CRF) = 28 was selected and used to control quality level of the HEVC encoded sequence. A set of 4 RSUs have been strategically located at 20, 300, 600, 1200 and 1500 m from-scene, and the distance between the RSUs and the road is 3 m. Notice that those RSUs are used just as traffic sinks to receive the video warning messages, in order to be able to measure the quality of the received video at several fixed distances from the incident. Figure 3a shows the map section considered, where buildings represented by pink rectangles are radio obstacles. This segment has an area of 2.5 km × 2.5 km and was retrieved from OpenStreetMaps [35]. Shadowing models are used to reproduce the attenuation of a radio signal induced by obstacles, such as buildings or other vehicles blocking the direct line of sight.

### 6.4. Performance Measures

In this article, we use four metrics to evaluate our two message dissemination protocols:

- Packet Delivery Ratio (PDR): It indicates the percentage of vehicles that received a single emergency message within a specified period of time *T*. We set T=30 s in our evaluations.
- Average Packet Delay (APD): It provides the average time from creating a message until it is finally received by the destination node.
- Broadcast Overhead (BO): It is measured as the number of global duplicate packets in a defined area.
- Number of collision packets (NCP): It is measured as the amount of packet collisions into the network topology during the data dissemination.

Additionally, we use two performance metrics to evaluate the quality of the video received:

- Frame Delivery Ratio (FDR): It is defined as the ratio between the number of frames delivered and the total number of frames received during a time interval T=120 s.
- Peak Signal-to-Noise Ratio (PSNR): It is an objective metric used to assess the application-level QoS of video transmissions. PSNR measures the error between the reconstructed image and the original one, frame by frame.

### 6.5. Simulation Results for Text Message Dissemination

In this section, we present some representative simulation results after a performance evaluation of our ADDs proposals compared to other approaches. Our goal is to study the capability dissemination of our proposal ADD under realistic urban scenarios. To do so, we have implemented the code of ADD with both the Volunteer’s Dilemma and the Forwarding Game as selection mechanisms of the next forwarding vehicle or vehicles. The purpose of the performance evaluation was to compare ADD with three well-known state-of-the-art protocols: Junction Store and Forward (JSF) [40], Neighbor Store and Forward (NSF) [9], RCP+ [30] and a simple flooding approach (Distance-Flooding) [12], in light of a realistic simulation environment.

- Junction Store and Forward (JSF) [40]: JSF is a protocol designed to exploit the road topology by considering that vehicles rebroadcasts the message every time they arrive at a new junction until the message timer expires. According to the JSF protocol, vehicles can store warning messages until a better communicating situation arises. This scheme requires each vehicle to maintain a neighbors’ table, which is updated taking advantage of the beacons exchanged by the vehicles. In adition, vehicles are assumed to use the information provided by the GPS to decide if a vehicle is near an intersection.
- Neighbor Store and Forward (NSF) [9]: NSF protocol was designed to tackle low density conditions. The behaviour of NSF is the following: after receiving a warning message, the vehicle waits until it finds a new neighbor to rebroadcast the message, that is, until it receives a beacon from another vehicle which is not contained in the neighbors’ table.
- Road Casting Protocol (RCP+) [30]: It is an efficient delay-based forwarding mechanism. It selects a set of forwarders with regard to the distances between the sender, the forwarder and the intersections; in addition, the link quality is estimated by means of channel quality, signal quality, and collision probability.
- Flooding-Distance [12]: This scheme relies on the concept of every vehicle having an internal counter of the number of times that a certain packet is received. The parameters employed by this algorithm are: the number of copies (*C*) that a node should hear a message to stop rebroadcasting that message, the maximum time ( MaxTime ) to rebroadcast and the shortest value between the distances to the original sending node ( OriginalDistance ) and the re-broadcaster node ( RebroadcasterDistance ). All optimal values for the urban scenarios are presented in Table 4.

**Figure 4:**
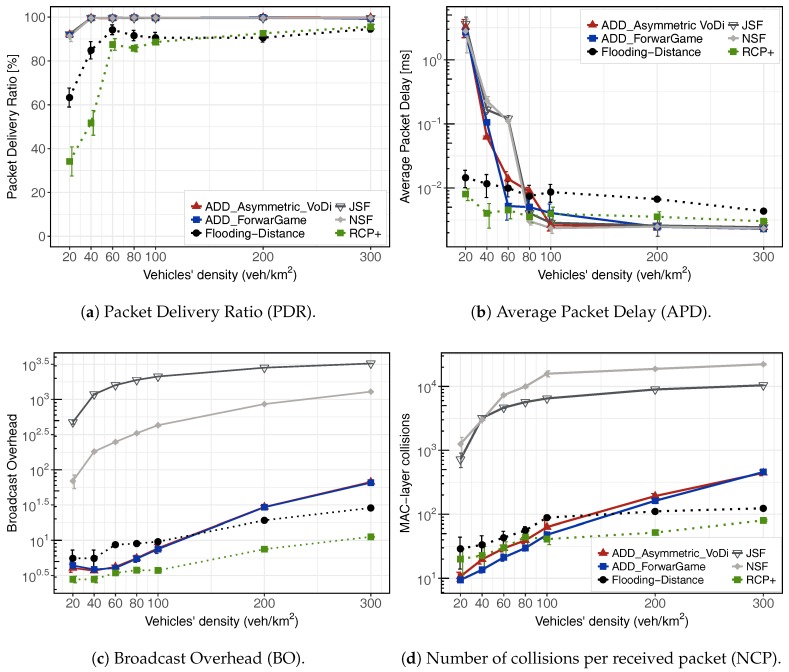
Results with 95% confidence intervals for 10 repetitions per point with independent seeds. Text dissemination case. Different vehicles’ densities in a 2.5 km × 2.5 km urban region in Berlin, Germany.

Figure 4 shows the results for an urban scenario when varying the network density from 20 to 300 veh./km ${}^{2}$ . First, we evaluate the global effectiveness of our solutions. We consider that the dissemination protocol is effective if it is able to deliver the information about the emergency event to all vehicles before time period expires. The time of the packet delivery for various VANET applications is defined in [41]. Figure 4a shows the packet delivery ratio of all aforementioned protocols. ADD, JSF and NSF achieve near 100% in delivery ratio for densities higher than 40 veh./km ${}^{2}$ . Notice that ADD-Forwarding Game and ADD-Volunteer’s Dilemma have a high performance for high traffic scenarios. This result was expected, since both protocols were designed to mitigate the broadcast storm problem. In contrast, RCP+ and Distance-Flooding present lower delivery ratio, even in high densities. In a low traffic scenario (20 veh./km ${}^{2}$ ), ADD, JSF and NSF schemes deliver the message to about 90% of the vehicles. On the other hand, RCP+ and Distance-Flooding present lower delivery ratio. This is fundamentally because, at the moment that an emergency message is generated, there might be no vehicle in the neighborhood to receive and disseminate the message to other vehicles on the road. Nevertheless, both ADD, NSF and JSF protocols present an improvement of near 45% in very low densities in terms of PDR, compared to RCP+ and Distance-Flooding schemes. This is explained by the fact that these protocols lack a SCF forwarding. Thus, nodes can not replicate the packet copies when the message has never been forwarded.

Moreover, the end-to-end delay shown in Figure 4b is the average delay it takes to disseminate a data packet from the source to all vehicles within the area of interest. In terms of end-to-end delay, SCF mechanism of ADD, JSF and NSF protocols incur longer delay for some messages compared to RCP+ and Distance-Flooding schemes when varying the network density from 20 to 60 veh./km ${}^{2}$ . This is explained by the fact that in low densities, ADD, JSF and NSF protocols have to frequently resort to using their SCF mechanisms. Thus, their performance in terms of end-to-end delay and delivery ratio becomes dependent on the movement of nodes. The increase in end-to-end delay in ADD-Forwarding Game and ADD-Volunteer’s Dilemma are due to the scheduling, the waiting time of 5 ms required before contending with other nodes for re-transmission at each hop and mainly to the Store-Carry-Cooperative Forward (SCCF) module.

As the traffic density increases from 80 to 300 veh./km ${}^{2}$ , all protocols show the lowest delay since they do not have to resort to using their SCF mechanisms. Besides, we see how all schemes are far below the 100 ms delay limit requirement defined in [42] for safety messages dissemination. This shows that ADD is able to quickly disseminate messages whenever there exists end-to-end connectivity to one of the fixed vehicles responsible for gathering data messages.

Finally, the overhead and collision metrics allow us to assess the efficiency of our porposal. Because a high number of transmissions could lead to overload the network unnecessarily, RCP+, Distance-Flooding, ADD-Forwarding Game and ADD-Volunteer’s Dilemma protocols were designed to minimize the number of message transmissions in the network. As shown in Figure 4c, both ADD approaches, RCP+ and Distance-Flooding strongly decrease the number of messages exchanged, providing better results than JSF and NSF. Note that the lack of the SCF module in the RCP+ and Flooding-Distance schemes produces a low overhead. With our dissemination mechanisms, the number of messages decreases after a few seconds because when informed vehicles receive a beacon from an uniformed vehicle, they use SCCF mechanism to coordinate the rebroadcast of the message, thus avoiding redundant retransmissions. On the contrary, nodes with JSF or NSF will try to replicate the packet to all the neighboring nodes it encountered. Therefore, massive packet replications will impose a serious overhead. This overhead is not significant at low densities, although it could become a problem in scenarios with high vehicle densities. In general, JSF and NSF schemes efficiently disseminate messages in both dense and sparse vehicular networks. More specifically, they achieve a high delivery ratio with a low propagation delay in case of text dissemination, although both introduce excessive load in the network. Alternatively, ADD is a cross layer dissemination protocol capable to alleviate load by means of optimizing the packet forwarding mechanism. We also examined the performance of the wireless channel by measuring the number of collisions per received packet, which are depicted in Figure 4d on a log-scale. Adaptive beaconing always leads to a moderated number of collisions. However, the number of collisions caused by a static beaconing exponentially increases with the number of nodes in the network. In Section 6.6, we evaluate the performance of the adaptive beaconing module.

### 6.6. Simulation Results for Adaptive Beaconing

**Figure 5:**
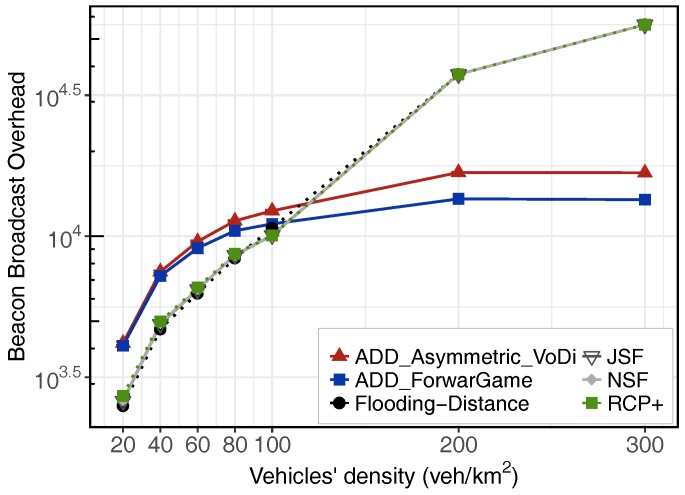
Beacon Overhead.

Exchanging vehicle information via beacon messages is an important feature for all schemes. All these protocols need beacon messages to discover neighbors and share local information. However, due to the beaconing periodic transmission, a substantially high load may be caused in the wireless channel. Our ADD proposals include an adaptive beaconing module, whereas RCP+, Distance-Flooding, JSF and NSF use a static beaconing scheme. ADD’s limits are configured accordingly to $I_{min}=30$ ms and $I_{max}=10$ s while the beaconing period of JSF and NSF is set to a traditional 1 s. Figure 5 depicts the effects of adapting the beacon rate in our proposal. According to the beacon overhead obtained, Distance-Flooding scheme, RCP+, JSF and NSF protocols introduce a lower overhead in the network for low to medium vehicles’ density, from 20 to 80 veh./km ${}^{2}$ . Both ADD schemes perform better under high vehicular density scenarios (in the interval between 100 and 300 veh./km ${}^{2}$ ). Concluding, adaptive beaconing can reduce significantly the number of beacons. Nonetheless, it is important to take into account that beacon sizes depend on the type and purpose of protocols. While beacons in Distance-Flooding, RCP+, JSF and NSF are considered as small packets periodically broadcast, the size of the beacon in ADD vary depending on the amount of data carried. In fact, beacons used by ADD contains a list of packet identifiers and this beacon could be notably large when there are a lot of packets being sent in the network. This could lead to an unpredictable behavior in the network and it could cause a scalability problem. To avoid this problem, in [43] authors proposed an efficient beacon solution that uses a Bloom filter. For that reason, we also plan to design a specific Bloom filter to represent the data inside of beacons as pointed out in Section 7.

### 6.7. Simulation Results for Video Warning Message Dissemination

**Figure 6:**
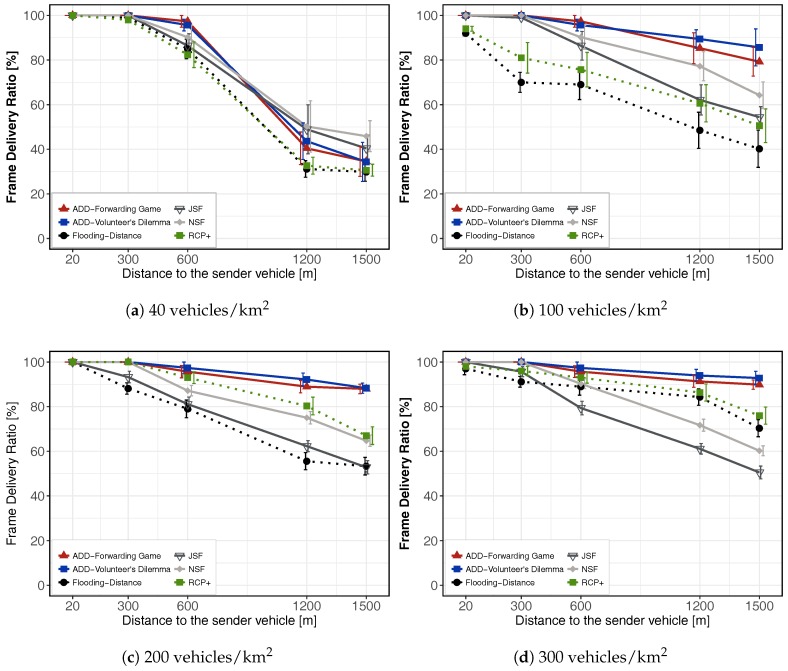
Frame Delivery Ratio (FDR) with 95% confidence intervals for 10 repetitions per point with independent seeds. Video dissemination case. Different vehicles’ densities in a 2.5 km × 2.5 km urban region in Berlin, Germany.

In this section, we present some representative simulation results for video content dissemination. Our goal is to study the dissemination capability of ADD under urban realistic scenarios. As seen previously, a video sequence is composed of I-, P-, and B-frames. We have evaluated the performance of the Frame Delivery Ratio (FDR), that is, the rate in which video frames are successfully delivered to each destination. Figure 6a shows the FDR for light vehicle density 40 veh./km ${}^{2}$ . Low density of vehicles directly affects the ability of the protocols to disseminate through the VANET. In fact, only $RSU_{1}$ (20 m) and $RSU_{2}$ (300 m) from the accident, received the complete trace. At $RSU_{3}$ located 600 m from the accident, ADD reaches a FDR of 97% and 95% with forwarding game and volunteer’s dilemma, respectively. In the NSF, JSF, Flooding-Distance and RCP+ schemes, we obtain an FDR of 90%, 86%, 84% and 82%, respectively. At the RSUs located at 1200 and 1500 m, all the protocols keep a FDR below 50% of received frames. This result is expected, because at the moment that a video packet is generated, there are cases where no vehicle is in neighborhood to receive and disseminate the video packet to other vehicles around. In addition, it is known that an urban scenario suffers more difficulties in the packet loss due to the existence of buildings. This causes temporary disconnections, interrupts the dissemination and compromises the delivery of the frames.

Figure 6b shows the FDR for 100 veh./km ${}^{2}$ . Moderated density of vehicles improves the FDR. This is evident in $RSU_{3}\;(600$ m), $RSU_{4}\;(1200$ m) and $RSU_{5}\;(1500$ m) where the FDR increases with respect to Figure 6a for all the tested schemes. For instance, at $RSU_{3}$ located 600 m from the accident, ADD reaches an average maximum rate of 97% and 95% with forwarding game and volunteer’s dilemma, respectively, while in the JSF, NSF, Flooding-Distance and RCP+ schemes, we obtain an FDR of 86%, 90%, 69% and 75%, respectively. At the RSUs located at 1500 m, JSF, NSF, Flooding-Distance and RCP+ schemes keep a FDR below 64% of received frames. Conversely, ADD reaches an average maximum rate of 79% and 85% with forwarding game and volunteer’s dilemma, respectively. Here, we can notice how the game-theoretical schemes allow us to achieve a better performance in comparison to the other schemes. Figure 6c shows the frame delivery rate for a heavy vehicles’ density (200 veh./km ${}^{2}$ ). In all RSUs, ADD-Forwarding Game and ADD-Volunteers’ Dilemma schemes reach levels above 88% of received frames. We can see that most schemes are able to provide more than 78% of the FDR at a distance of 600 m from the accident. We can also notice that ADD is able to improve the FDR between 1200 m and 1500 m. This is due to the reduced number of collisions produced when ADD is used. Figure 6d shows the FDR for high vehicles density (300 veh./km ${}^{2}$ ). A traffic jam situation directly affects the ability of JSF and NSF schemes to disseminate video messages, since in this scenario, the number of collisions increases exponentially as it can be seen in the Figure 6d. This retrains the progress of the packets and consequently the information will reach closer vehicles only. This can be seen at $RSU_{4}(1200$ m) and $RSU_{5}(1500$ m) where the FDR does not exceed 60% of received frames despite a high connectivity in the network. It is important to highlight that JSF and NSF protocols were designed for the effective dissemination of text messages at low vehicles’ densities [17]. However, these same characteristics that make them successful disseminators in low densities end up affecting their performance in high densities. While JSF resends video messages in an unlimited number of junctions, NSF resends video messages each time it finds a new neighbor. We can also notice that RCP+ and Distance-Flooding are able to improve the packet delivery ratio. This is due to the reduced number of collisions produced when these schemes are used. In all RSUs, ADD-Forwarding Game and ADD-Volunteers’ Dilemma schemes are able to deliver more than a 90% of the frames at a distance as far as 1500m. With our dissemination mechanisms, the selection of potential forwarders is controlled by a game-theoretical algorithm (see Section 3). When the network is partitioned due to low vehicles’ density, the SCCF module coordinates the selective forwarding only when informed vehicles receive a beacon from an uniformed vehicle (see Algorithm 3, lines 1–6), thus avoiding redundant retransmissions.

**Figure 7:**
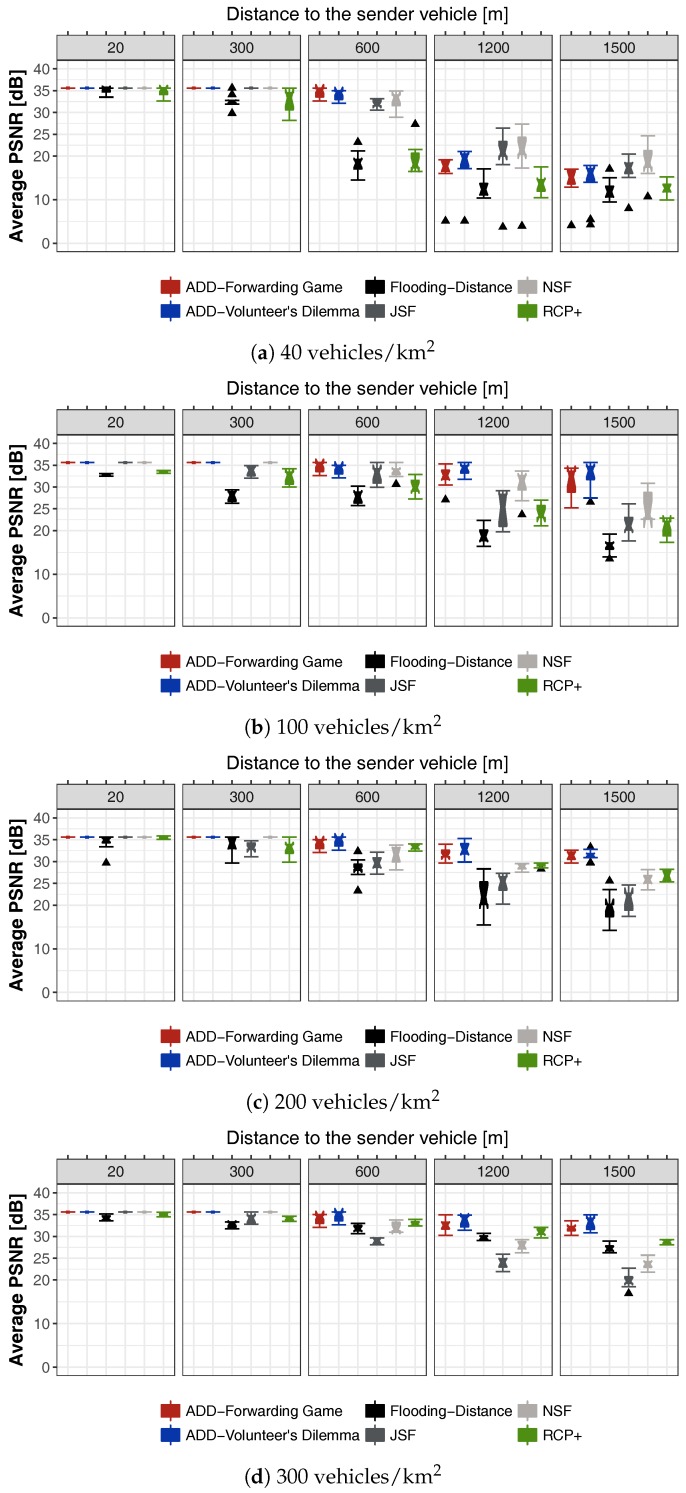
PSNR for video dissemination with 95% confidence intervals for 10 repetitions per point with independent seeds. Different network densities in a 2.5 km × 2.5 km urban region in Berlin, Germany.

As a next step, we have evaluated the quality of a received video in terms of the Peak Signal to Noise Ratio (PSNR). We assume that in the case an individual video frame was lost, the decoder would replace that lost frame by the last successfully received frame (of same type) instead. Thus, if a frame is dropped, we need to compare the source frame to the previous received frame of the same type. Next, we decoded each frame into its YUV channels. The PSNR of the channels need to be calculated independently. We just use the Y (luminance) channel, since the human eye is far more sensitive to the presence of noise and distorsions in brightness rather than the presence of errors and distorsions in the color [44]. According to a classification presented in [45], PSNR values higher than 37 dB, guarantee an excellent video quality on the receiver side. If this value varies between 31 dB and 37 dB, the received video quality will be good. When PSNR values varies between 25 dB and 31 dB we have a fair video quality on the receiver side. If PSNR is lower than 25 dB it provides poor video quality to users. Figure 7 shows the average PSNR of the reconstructed video at the receivers’ vehicles in RSUs located at 20, 300, 600, 1200 and 1500 m for different traffic densities. These results show how distance and traffic congestion affect video performance at each RSU. Figure 7a shows the average PSNR for low vehicles density (40 veh./km ${}^{2}$ )). Low vehicles’ density directly affects the ability of the protocols to disseminate messages. We can see that the average PSNR in the game-theoretical schemes are all higher than 35 dB (good video quality) in RSUs locate at 20 and 300 m. In the RSUs located at 1200 and 1500 m. all the protocols keep a PSNR below 25 dB. This poor quality is caused by temporary disconnections which provoke long loss bursts. As traffic density increases (see Figure 7c,d), we see how the quality of the received video presents a growing trend in all the protocols.

**Figure 8:**
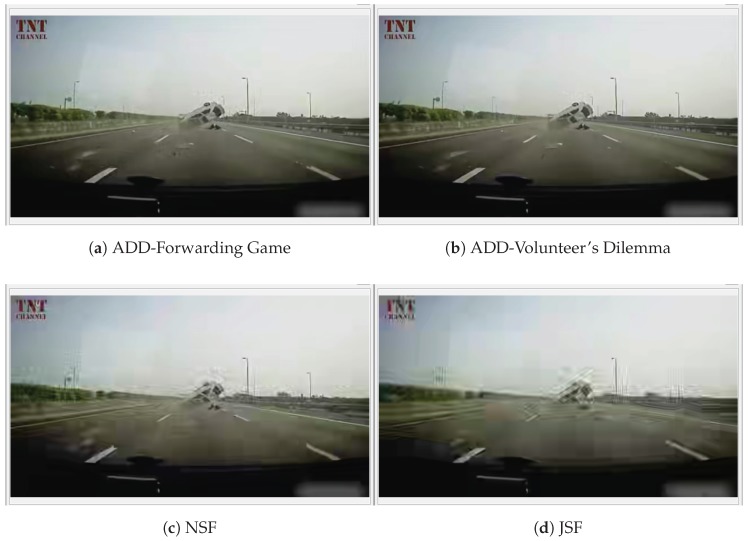
Comparison sample for the different simulated protocols at frame 72 in $RSU_{4}$ located at 1200 m with 100 vehicles/km ${}^{2}$ .

As illustrated in Figure 7d, ADD-Forwarding Game and ADD-Volunteer’s Dilemma provide good to excellent video quality (PSNR > 31) in all RSUs. On the other hand, RCP+, Distance-Flooding, JSF and NSF schemes provide fair to good video quality (25 < PSNR < 37) in RSUs locate at 20, 300, 600 m. In $RSU_{4}$ located at 1200 m and $RSU_{5}$ located at 1500 m, the average PSNR in JSF and NSF schemes provide poor video quality (PSNR < 25 dB). Despite the good performance of both JSF and NSF schemes in the dissemination of text messages, when we send video messages we observe a poor performance. The reason is that those schemes, which were not specially designed for video dissemination, generate excessive redundant transmissions, which may lead to a broadcast storm problem. In those situations, the network suffers from the increasing administrative load, especially as the number of vehicle nodes increases. Likewise, RCP+ and Distance-Flooding provide fair video quality (25 < PSNR < 30). This performance mainly occurs because the video stream suffers from loss bursts associated with the protocol’s difficulty to maintain the dissemination and thus compromising the delivery of the video packets. Another interesting observation is that the calculated confidence intervals are quite large which indicates that results vary significantly. The reason for this is that, although all video packets are treated in the same way, they contain the information of different frames (I and P frames). This information has a different impact on the overall video quality. We randomly selected one sample frame from the transmitted video, aiming to give the reader an idea of the user’s point-of-view, as illustrated in Figure 8. Frame 72 is the moment when a person is thrown out of the vehicle. The transmitted sequences using ADD have low distortion compared to the same frame sent using JSF and NSF. This is mainly because ADD-Forwarding Game and ADD-Volunteer’s Dilemma schemes do not only rely on the distance factor information, but also consider the position of the vehicle in the network (distance between receiver to next junction, distance between transmitter and receiver), an estimation of the link quality (signal quality, channel quality and collision probability), and an estimation of the available bandwidth. All these factors taken into account improve the video dissemination performance.

In general, video dissemination is a demanding task for any kind of network because of high bandwidth utilization and strict delay requirements. Furthermore, VANETs provide one of the most difficult environments to achieve a good video transmission quality. Results show that ADD is clearly able to perform well in all the investigated scenarios. The comparison shows that ADD-Forwarding Game and ADD-Volunteer’s Dilemma are effective and efficient in video dissemination without incurring a high load into the network. In addition with the proposed ADD scheme, real-time video in VANET environments is feasible even in long distances (1500 m), if the density of vehicles on the road is moderated or jam (100–300 veh./km ${}^{2}$ ). On the other hand, when the vehicular density is low, video transmission is difficult in urban scenarios without a backhaul communication infrastructure. A combination of both protection of packets at network level and error resilience techniques at application level could be welcome to guarantee a high video quality.

## 7. Conclusions and Future Work

In this work, we have modeled a cooperative game where vehicles have the choice to participate in the data dissemination process or not. First, we have evaluated the use of the asymmetric volunteer’s dilemma game as a mechanism for mitigating the broadcast storm in VANETs. An optimized utility function based on distance and link quality was proposed for enhancing data dissemination. Additionally, we have developed a forwarding game where each vehicle has a utility that is a function of its own strategy (its forwarding probability), availability and of the strategy of its neighbors. In this game, an optimized availability function based on distance and an estimated bandwidth was proposed. The availability component of the utility function was designed to improve the network performance by eliminating redundant broadcasts. Both Volunteer’s Dilemma and Forward Game have been evaluated in terms of packet delivery ratio, average packet delay, broadcast overhead, and number of collision packets. In addition, we focus on a beaconing module that captures both beacon message priority and channel conditions to adapt to highly dynamic environments that change from fully connected to disconnected states. This adaptivity is achieved by nodes continuously sensing their surroundings in order to quickly and dynamically react to changes. In general, ADD selects a minimum set of vehicles to broadcast and also estimates when the broadcast should take place. This way, ADD protocol tries to reduce the load sent to the link layer by decreasing the amount of redundant re-transmissions. Moreover, given that network partitioning is very common in VANETs, independently of the traffic density, received messages are kept in a local buffer to be later forwarded to uninformed vehicles. Simulation results show that the proposed schemes can reduce broadcast overhead and collision packets while still offering acceptable end-to-end delay for most multihop VANET applications. The models developed here provide efficient mechanisms for mitigating the broadcast storm and insights into how vehicular networks can be a platform to develop cooperative communication systems. Future work includes to design a dynamic scheme to update the weights of the multimetric score to calculate the utility function $U_{i}$ for node *i* (see Equation (14)) so that the algorithm is self-configured and adapts to the changing environment. We will use machine learning techniques to attain this goal. In addition, we plan to extend the model to analyze the behavior of the nodes based on the benefits earned by player *i* when at least one player volunteers, as an awards strategy for enhancing cooperation. Finally, we will introduce a scalable proactive content discovery scheme, hierarchical bloom-filter routing, to tackle mobility, large population, and rich content challenges of VANETs.

## Figures and Tables

**Table 1:** Definitions of the variables presented in the Asymmetric Volunteer’s Dilemma.

Variable	Definition
$\beta_{i}$	Probability of defection of player *i*
$K_{i}$	Cost of volunteering for player *i*
$U_{i}$	Benefit earned by player *i* when at least one player volunteers
$\beta_{j}$	Average defection probability of all the other players *j* (j≠i)
$\beta_{i}^{N}$	Probability that nobody volunteers
*i* =1, 2, 3, …, *N*	*i* is a generic player, being *N* the number of players

**Table 2:** Definitions of the variables presented in the Forwarding Game.

Variable	Definition
$S_{i}$	Probability that node *i* forwards the received flooding packet
$U_{i}$	Utility of node *i*
$a_{i}$	Availability of node *i*
S_i	Average forwarding probability of the neighboring nodes of *i*
$Q_{i}$	Neighbor action reflection
k,m,n	Constant values. Our results showed that *k* = 4, *m* = 2, *n* = 3 provide optimum results based on our simulations.
*i* = 1, 2, 3, …, *N*	*i* is a generic node, being *N* the number of nodes

**Table 3:** Vehicle types and associated probability in urban scenarios. SUMO parameters.

Vehicle Type	Maximum Speed (m/s)	Lengh (m)	Height (m)	Probability (%)
Slow Car	14	5	2	5
Car	25	4	2	69
Fast Car	33	4	3	1
Bus	17	12	3.4	25

**Table 4:** Simulation parameters.

	Parameter	Value
**Physics and MAC Layers IEEE 802.11p**	Bandwidth	10 MHz
	Channel Frecuency	5.89 GHZ
	Transmission range	∼300 m. Defined in [36]
	Transmission power	10 mW
	Sensitivity	−89 dBm
	Obstacle model	Defined in [37,38]
	$AC_{BE}$ [CWmin, CWmax], AIFSN	[15,1023], 6
	$AC_{Video}$ [CWmin, CWmax], AIFSN	[7,15], 3
	Bit rate	6 Mbps
**ADD**	$RSS_{th}$ , $RSS_{max}$	‒89 dBm, ‒20 dBm
	Time slot	13 μ s
	Time window	10 s
	δ (Waiting Time)	[1,11]μ s
	Beacon frecuency	Defined in [5]
	Beacon size	> =32 B
	Data size	2312 B
	Video file size	5399 KB
	Video Codec	H.265/HEVC, yuv420p, 25 fps
		Low-Delay P (LP)
	Constant Rate Factor (CRF)	28
	Duration	1 min 20 s
	Video resolution	640 × 360
**Adaptive Beaconing**	$I_{min}$	30 ms
	$I_{max}$	10 s
	$W_{I}$	0.35
	$W_{C}$	0.5
**NJL** **NSF**	Warning message size	256 B
	Beacon Message size	512 B
	Warning messages priority	$AC_{3}$
	Beacon priority	$AC_{1}$
	Beacon frecuency	1 Hz (1 beacon per second)
**RCP+**	$RSS_{th}$ , $RSS_{max}$	−89 dBm, −20 dBm
	Time slot	13μ s
	Time window	10 s
	δ (Waiting Time)	[1,11]μ s
**Flooding-Distance**	MaxTime	500 ms
	Counter *C*	1 (80, 100, 200, 300 veh./km ${}^{2}$ )
		2 (60 veh./km ${}^{2}$ )
		3 (20, 40 veh./km ${}^{2}$ )
**Scenarios**	Number of Runs	10
	Time to live (TTL)	30 s (text), 120 s (video)
	Vehicles’ density	20, 40, 60, 80, 100, 200, 300 veh./km ${}^{2}$
	Area of interest to warn vehicles	2.5 km × 2.5 km

## Abbreviations

The following abbreviations are used in this manuscript:

ABE	available bandwidth estimation
ADD	adaptive distributed dissemination
AMD	adaptive multi-directional data dissemination
APAL	adaptive probability alert protocol
ATB	adaptive traffic beacon
BSM	basic safety message
CAM	cooperative awareness message
CJ	critical junction
CRF	constant rate factor
DV-CAST	distributed vehicular broadcast
FDR	frame delivery ratio
GPS	global positioning system
HEVC	high efficiency video coding
ITS	intelligent transport systems
JSF	junction store and forward
MAC	Medium access control
MANET	mobile ad-hoc network
NSF	neighbor store and forward
NJL	nearest junction located
PDR	Packet delivery ratio
PLR	packet loss ratio
PSNR	peak signal-to-noise ratio
QoS	quality of service
RCP	road casting protocol
ROI	region of interest
RSU	road side unit
SCF	store-carry-forward
SCCF	store-carry-cooperative forwarding
SNR	signal-to-noise ratio
SUMO	simulation of urban mobility
TraCI	traffic command interface
TTL	time to live
UDP	user datagram protocol
VANET	vehicular ad hoc network
VEINS	vehicles in network simulation
VoDi	volunteer’s dilemma
WAVE	wireless access in vehicular environments
WHO	world health organization
WSMP	wave short message protocol
